# Phosphoproteomic and proteomic profiling in post-infarction chronic heart failure

**DOI:** 10.3389/fphar.2023.1181622

**Published:** 2023-06-19

**Authors:** Jiayue Wang, Xiuhua Zhu, Shenrui Wang, Yingjie Zhang, Wenjie Hua, Zhenyu Liu, Yu Zheng, Xiao Lu

**Affiliations:** Department of Rehabilitation Medicine, The First Affiliated Hospital of Nanjing Medical University, Nanjing, China

**Keywords:** post-infarction chronic heart failure, phosphoproteomics, Bclaf1 Ser658, apoptosis, proteomics

## Abstract

**Background:** Post-infarction chronic heart failure is the most common type of heart failure. Patients with chronic heart failure show elevated morbidity and mortality with limited evidence-based therapies. Phosphoproteomic and proteomic analysis can provide insights regarding molecular mechanisms underlying post-infarction chronic heart failure and explore new therapeutic approaches.

**Methods and results:** Global quantitative phosphoproteomic and proteomic analysis of left ventricular tissues from post-infarction chronic heart failure rats were performed. A total of 33 differentially expressed phosphorylated proteins (DPPs) and 129 differentially expressed proteins were identified. Bioinformatic analysis indicated that DPPs were enriched mostly in nucleocytoplasmic transport and mRNA surveillance pathway. Bclaf1 Ser658 was identified after construction of Protein-Protein Interaction Network and intersection with Thanatos Apoptosis Database. Predicted Upstream Kinases of DPPs based on kinase-substrate enrichment analysis (KSEA) app showed 13 kinases enhanced in heart failure. Proteomic analysis showed marked changes in protein expression related to cardiac contractility and metabolism.

**Conclusion:** The present study marked phosphoproteomics and proteomics changes in post-infarction chronic heart failure. Bclaf1 Ser658 might play a critical role in apoptosis in heart failure. PRKAA1, PRKACA, and PAK1 might serve as potential therapeutic targets for post-infarction chronic heart failure.

## Introduction

With the aging of the global population, the prevalence of chronic heart failure continues to increase year by year and is projected to rise up to 3.0% in 2030 (Heidenreich et al., 2013; Tsao et al., 2022). Post-infarction chronic heart failure, developed by coronary artery disease with previous myocardial infarction (MI), is the most common type of heart failure (Townsend et al., 2022). Advances in treatments improved survival rates of acute MI patients (Nishihira et al., 2020). However, more survivors suffered from adverse post-infarction left ventricular (LV) remodeling and developed post-infarction chronic heart failure (Galli and Lombardi, 2016). Patients with chronic heart failure show elevated morbidity, mortality, and readmission rate (Dunlay et al., 2010) and currently available treatments are only capable of delaying the progression of heart failure (Nelson et al., 2011). Post-infarction chronic heart failure, as a multifactorial systemic disease, involves several activated regulatory mechanisms (Tanai and Frantz, 2015). Hence, there is an urgent need to investigate the molecular mechanisms underlying post-infarction chronic heart failure and to identify more targets for treatment.

Proteomics are powerful tools that allow for investigating complex biological processes at the protein level and are considered one of the most potent tools in biomedicine research (Reichl et al., 2020). Alterations in proteome often reflect disease aetiologies and mechanisms (Arrell et al., 2001). Previous studies have identified protein changes in heart failure with preserved ejection fraction, heart failure with dilated cardiomyopathy, and non-ischemic heart failure (Corbett et al., 1998; Schechter et al., 2014; Valero-Muñoz et al., 2022). These studies revealed differences in protein quantity in heart failure and provided a new sight into molecular mechanisms underlying heart failure. However, apart from differences in protein quantity, many of the key proteins involved in signal pathways are tightly connected to post-translational modifications (PTMs), such as phosphorylation (Steeg, 2003; Hu et al., 2020). Recently, Ercu et al., (2022) reported that mutant phosphodiesterase 3A (PDE3A) led to aberrant phosphorylation and altered adrenergic signaling pathway, which resulted in protection against hypertension-induced cardiac damage in hearts. Zhuang et al., (2022) demonstrated that dual-specificity tyrosine-regulated kinase 1B (DYRK1B) directly bound to signal transducer and activator of transcription 3 (STAT3) and increased its phosphorylation, leading to aggravating mitochondrial damage, cardiac hypertrophy, and heart failure. However, regulation of phosphorylation in post-infarction chronic heart failure remains poorly understood.

In this study, we conducted a combined quantitative phosphoproteomic and proteomic analysis of left ventricular (LV) tissues from post-infarction chronic heart failure rats. A total of 33 differentially expressed phosphorylated proteins (DPPs), 37 phosphorylation sites, and 129 differentially expressed proteins were identified. We then performed gene set enrichment analysis, functional annotation, protein-protein interaction (PPI) network construction, intersection of DPPs and Thanatos Apoptosis Database, validation of Bcl-2-associated transcription factor 1 (Bclaf1) Ser658, and predictions of upstream kinases. Proteomic analysis showed marked changes in protein expression related to cardiac contractility and metabolism. In summary, we revealed distinct protein phosphorylation patterns during post-infarction chronic heart failure and these results might support the development of efficient treatments for post-infarction chronic heart failure.

## Methods and materials

### Animal model of chronic heart failure

Healthy male Sprague-Dawley (SD) rats, specific-pathogen-free (SPF) grade, weighing 180–220 g, were obtained from Beijing Vital River Laboratory Animal Technology Co., Each rat was housed in a regular environment of 12 h light/dark cycle with free access to water and food for 1 week in the Animal Experimental Center of Nanjing Medical University. The SD rats were randomly divided into the sham operation (SO) group and the HF group. Under anesthesia, a thoracotomy was performed through the fourth intercostal space, the heart was exposed. A suture was placed 1–1.5 mm from the left anterior descending branch (LAD), and the ends were tied loosely in the SO group and firmly in the HF group. The rat model of post-infarction chronic heart failure was confirmed with echocardiography (mainly due to LV ejection fraction lower than 50%) after 4 weeks of feeding. Left ventricular internal diameter at diastole (LVID; d), LV end internal diameter at systole (LVID; s) and fractional shortening (FS) were also measured and calculated. After echocardiography, two groups (HF, N = 3; SO, N = 3) were sacrificed under anesthesia by cervical decapitation. Serum and heart tissue were collected.

### Masson assay

The detection of fibrosis within heart tissue samples was carried out utilizing a Masson assay. 10% buffered formalin was used to fix fresh heart tissues. The fixed tissues were then embedded in paraffin and cut into sections with a thickness of 5 μm. These sections were subsequently stained with Masson’s trichrome and examined under a microscope. In each section, eight random sites were selected to measure the fibrotic area. The analysis of each section was performed using an HMIAS Series Color Medical Image Analyze System (Champion Image Ltd., China).

### Serum brain natriuretic peptide assay

The brain natriuretic peptide (BNP) is a biomarker of CHF used to determine the severity of the condition. The serum concentration of BNP was detected using ELISA kits in accordance with the manufacturers’ instructions (BNP Elisa Kit: Sigma, Saint Louis, United States).

### Peptide labeling and TiO_2_ enrichment

Peptide labeling of the infarct margin zone of heart was performed with a tandem mass tags (TMT) kit (Thermo Fisher Scientific) following the manufacturer’s protocol. Dithiothreitol (DTT) was added to 100 μg proteins to a final concentration of 10 mM. Mixtures were incubated at room temperature for 1 h and then added by iodoacetamide (IAM) to a final concentration of 40 mM. Peptides were digested with 100 μg trypsin. One unit of TMT reagent was thawed and dissolved in 41 μL acetonitrile. The peptide mixtures were then incubated at room temperature for 1 h and pooled, and dried by vacuum centrifugation using an Eppendorf Concentrator Plus.

Phosphopeptide enrichment using TiO_2_ chromatography was performed. The trypsin-digested peptides were enriched for phosphopeptides using Titansphere TiO_2_ tips (Titansphere). Phosphopeptides were serially eluted in 5% NH_4_OH in water, 5% pyrrolidine in acetonitrile, and 60% acetonitrile in water. The three elutions were pooled together and neutralized with 50% acetic acid. Tryptic peptides were separated on a C18 column with an acetonitrile gradient (6, 9, 12, 15, 18, 21, 25, 30, and 35%), and dried. Samples were reconstituted in 50 μL 0.03% Trifluoracetic acid (TFA, ACROS). Each enriched sample was desalted using a Stage Tip (Thermo Fisher Scientific) per the vendor protocol. Peptides were dried and reconstituted in 70 μL of 0.03% TFA prior to analysis.

### LC-MS/MS analysis

Peptides were then analyzed using liquid chromatography and dual mass spectroscopy (LC-MS/MS) on an Orbitrap Fusion Lumos mass spectrometer (Beijing Puyuanjing) utilizing a binary solvent system. We prepared mobile phase solution A (0.1% formic acid in 100% MS water) and solution B (0.1% formic acid in 90% acetonitrile). Peptides were dissolved in 10 μL solution A and loaded on an ACQUITY UPLC PST (BEH) C18 nanoACQUITY Column (75 μm inner diameter). The mixture was eluted at 600 nL/min with the following gradient: 7% buffer B at initial conditions; 15% B in 11 min; 25% B in 48 min; and 25%–40% B for 48–65 min; and 100% B for last 10 min before returning to initial conditions.

Full MS scan was performed in profile mode over the 407–1,500 m/z scan range using 1 microscan, 120,000 resolution, AGC target of 4  ×  10^5^, and a maximum injection time (IT) of 50 ms. Data-dependent MS/MS scan was performed in centroid mode using 1 microscan, 50,000 resolution, AGC target of 5  × 10^3^, a maximum IT of 86 ms. The electrospray voltage applied was 2.0 kV. A data-dependent procedure that alternated between one MS scan followed by 10 MS/MS scans with 30.0 s dynamic exclusion was then applied.

UniProt protein database of *Rattus norvegicus* was used for raw MS/MS data search (Jiang et al., 2016; Wang et al., 2021). Calculation of the false positive rate (FPR) was performed with a reverse decoy database. Trypsin/P was specified as the cleavage enzyme allowing no more than 2 missing cleavages. The minimum length of peptides was set to 7 aa and the maximum number of modification sites was set to 5. During the first search, the mass tolerance for precursor ions was set to 20 ppm, whereas 5 ppm in the main search. The mass tolerance for fragment ions was set to 0.02 Da. Phosphorylation on Ser/Thr/Tyr was specified as variable modifications. The quantitative method was set as TMT-10plex. Protein identification and the FDR of the identified peptide spectrum match (PSM) were adjusted to <1%. The localization probability of site modifications was filtered with a value of >0.75.

### Gene set enrichment analysis in HF

Gene set enrichment analysis (GSEA) software (4.2.3) was employed to detect the related gene enrichment in HF. The significant gene sets that conform to the nominal (NOM) *p*-value <0.05 and false discovery rate (FDR) < 25% were shown.

### Functional annotation and pathway enrichment of DPPs

Gene Ontology (GO) biological process and Kyoto Encyclopedia of Genes and Genomes (KEGG) annotation were performed using the Bioinformatics website (http://www.bioinformatics.com.cn/). Biological process and KEGG pathway enrichment analyses using the “ClusterProfiler” package were performed to obtain insights into the potential functions of the DPPs. The top 10 results were shown in the enrichment scatter plots.

### Construction of protein-protein interaction network of DPPs

The STRING database (http://string-db.org/) was employed to analyze the interactions of the distinct DPPs. Cytoscape software 3.9.1 (http://cytoscape.org/) was then utilized to construct and visualize the protein-protein interaction (PPI) network. The molecular complexes were examined using the MCODE algorithm. The top 6 DPPs of the PPI network were defined as the hub DPPs, which were calculated based on the maximum neighborhood component (MNC), degree, and edge percolated component (EPC) algorithms by utilizing the cytoHubba plug-in.

### Intersection of DPPs and thanatos apoptosis database

Cardiomyocyte apoptosis and fibrosis after myocardial infarction serve as essential roles in the formation and progression of heart failure (Tanai and Frantz, 2015; Federico et al., 2017). Thus, investigations of critical regulators of cardiomyocyte apoptosis are essential for the treatment and prevention of heart failure. Thanatos Database (http://thanatos.biocuckoo.org/index.php#) collected more than 4,000 experimentally identified proteins regulated in cell death pathways (Deng et al., 2018), including autophagy, necrosis, and apoptosis. In this study, the intersection of DPPs and Thanatos Apoptosis Database was analyzed through Venn Diagram. Subsequently, the result of intersection was analyzed with 6 hub DPPs.

### Kinome profiling

Kinome profiling was conducted using the phosphoproteomic data and the kinase-substrate enrichment analysis (KSEA) app (Wiredja et al., 2017). Results of KSEA with *p* values less than 0.05 was used in this study.

### Western blotting

Mechanically homogenized frozen rat LV tissues (8 rats, 4 in each group) were lysed in RIPA buffer (Biyuntian) with protease and phosphatase inhibitors (Biyuntian) on ice for 30 min. Protein concentration was determined with BCA assay (Biyuntian). Equal levels of protein (30–50 μg) were subjected to electrophoresis and blotted to polyvinylidene difluoride (PVDF) membranes. The membranes were blocked in 5% bovine serum albumin (BSA) for 2 h at room temperature and then incubated for 8 h at 4°C with rabbit Bclaf1 Ser658 antibody (Abmart), protein kinase catalytic subunit alpha-1 gene (PRKAA1) antibody (Proteintech), protein kinase A catalytic α subunit (PRKACA) antibody (Proteintech), protein kinase D1 (PKD1) antibody (Immunoway), p21-activated kinase 1 (PAK1) antibody (Immunoway), protein kinase C delta (PRKCD) antibody (Proteintech), and glyceraldehyde 3–phosphate dehydrogenase (GAPDH) antibody (Abmart). The membranes were then washed with Tris-Buffered Saline Tween-20 (TBST) and incubated for 2 h at room temperature with anti-rabbit secondary antibody (Abmart). Results were visualized by Tanon-410 automatic gel imaging system (Shanghai Tianneng).

### Statistical analysis

Statistical analysis was performed using the GraphPad Prism software version 8.0. Data are presented as the mean ± Standard Error of Mean (SEM) from at least two independent experiments. Differences in measured variables between HF and SO groups were assessed using the unpaired Student’s *t*-test. *p* values were calculated and minimum statistical significance was accepted at *p* < 0.05.

## Results

### Rat models of post-infarction chronic heart failure

**FIGURE 1:**
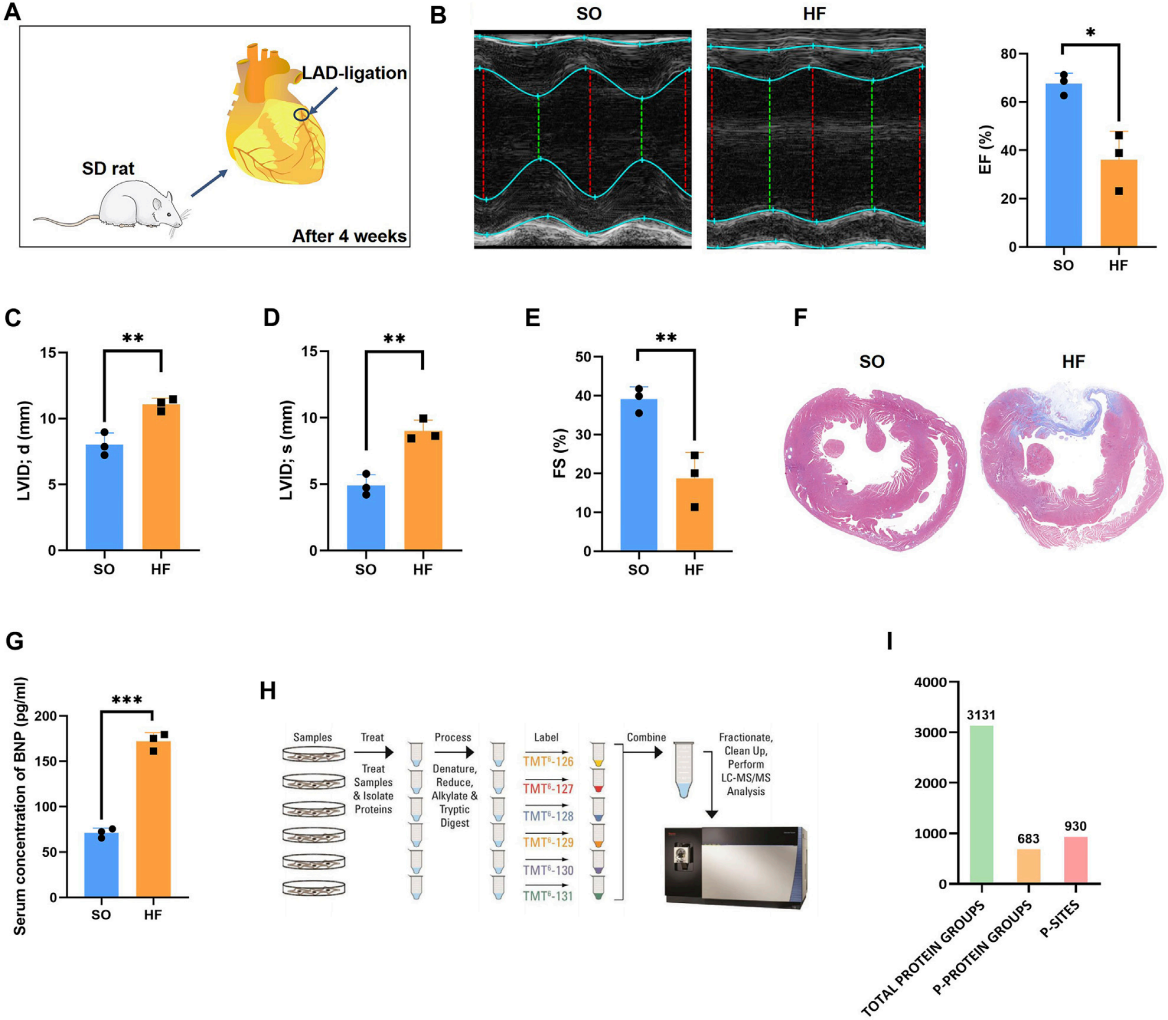
Phosphoproteomics workflow **(A)** Rat models of post-infarction chronic heart failure created by permanent ligation of the left anterior descending (LAD) and raising for 4 weeks. **(B)** Representative echocardiography and the mean left ventricular ejection fraction (EF) of rats in HF and SO group. **(C)** The mean left ventricular internal diameter at diastole (LVID; d) of rats in HF and SO group. **(D)** The mean left ventricular end internal diameter at systole (LVID; s) of rats in HF and SO group. **(E)** The mean fractional shortening (FS) of rats in HF and SO group. **(F)** Comparison of fibrosis within heart tissue between two groups. **(G)** Comparison of the serum BNP concentration between two groups. **(H)** Work flow of phosphoproteomic analysis **(I)** Amount of identified total peptides, total protein groups, phosphorylated proteins, and phosphor-sites.

As previously described (Zhang et al., 2021a), post-infarction chronic heart failure was induced in adult rats by permanent ligation of left anterior descending coronary artery (Figure 1A). Four weeks after surgery, compared to SO group, rats in HF group demonstrated a decrease in LV ejection fraction (67.57% ± 4.40% vs. 36.10% ± 11.78%, *p* < 0.05; Figure 1B). In addition, in HF group, LVID; d and LVID; s increased significantly (*p* < 0.01; Figures 1C, D) and FS decreased significantly (*p* < 0.01; Figure 1E) compared with SO group. The Masson assay showed that HF induced remarkable fibrosis in the myocardial tissues of SD rats (Figure 1F). The serum concentration of BNP in HF group was significantly higher compared with SO group (*p* < 0.001; Figure 1G).

### Phosphoproteomic profiling of the LV in post-infarction chronic heart failure

**FIGURE 2:**
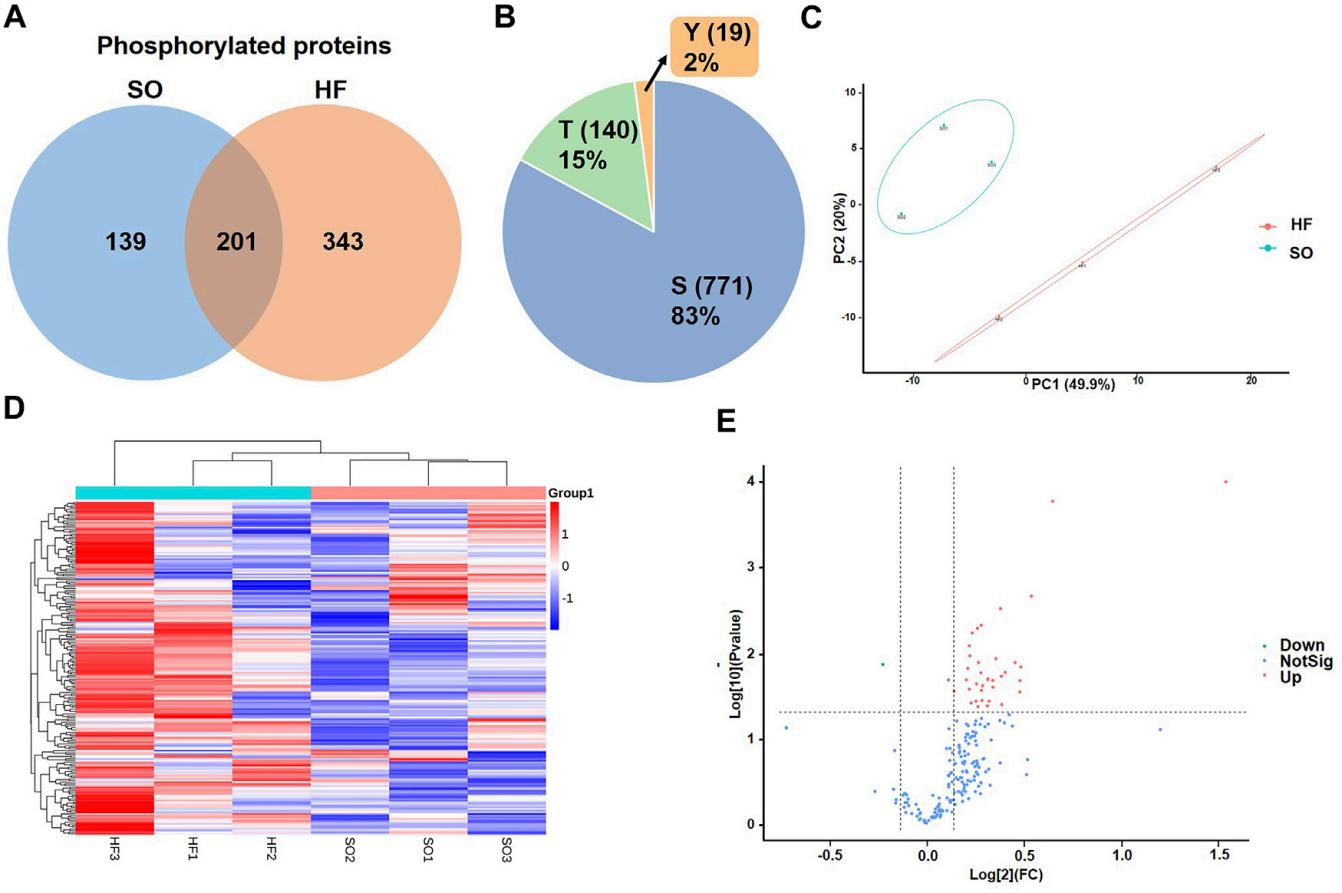
Identification of differentially expressed phosphorylated proteins **(A)** Venn diagram of phosphorylated proteins in HF and SO group. **(B)** Distribution of serine (S), threonine (T), and tyrosine (Y) phosphorylation based on differential phosphor-sites. **(C)** PCA analysis of HF group and SO group. The ordinate and abscissa represent the first principal component and the second principal component, respectively. Each dot in this figure represents a sample, and samples in the same group are represented by the same color. **(D)** Heat map comparison of the post-infarction chronic heart failure (HF) group and sham operation (SO) group. Each column is a sample and each row is a phosphorylated protein with phosphor-site. HF group compared to SO group: Red represents upregulated phosphorylated proteins and blue represents downregulated phosphorylated proteins. **(E)** Volcano plots displaying significantly differentially expressed phosphorylated proteins. Red dots represent the upregulated phosphorylated proteins and blue dot represents the downregulated phosphorylated protein.

In this study, a combined quantitative phosphoproteomic and proteomic analysis was performed in LV tissues obtained from 3 post-infarction chronic heart failure rats and 3 respective SO (Figure 1H). A total of 3,131 proteins, 683 phosphorylated proteins, and 930 phospho-sites were identified and then used for analysis (Figure 1I). Among 683 identified phosphorylated proteins, 201 phosphorylated proteins were expressed both in post-infarction chronic heart failure and SO groups (Figure 2A). Among 930 phospho-sites, we identified 771, 140, and 19 phosphorylated serine, threonine, and tyrosine residues, respectively (Figure 2B).

We then used correspondence analysis to visualize the discrepancy between HF and SO groups. Principal component analysis (PCA) model established in this study showed that HF group was completely separated from SO group (Figure 2C), indicating that when compared with normal LV tissues, LV tissues from post-infarction chronic heart failure were significantly altered in total phosphorylation level.

We identified a total of 33 phosphorylated proteins and 37 phosphorylation sites that varied between the HF and SO groups (fold change cutoff >1.1, *p* < 0.05; Figures 2D, E). Remarkably, only one differentially expressed phosphorylated protein was downregulated in the HF group and more than 90% (34 phospho-sites) were serine (Supplementary Table S1).

### Gene set enrichment analysis

**FIGURE 3:**
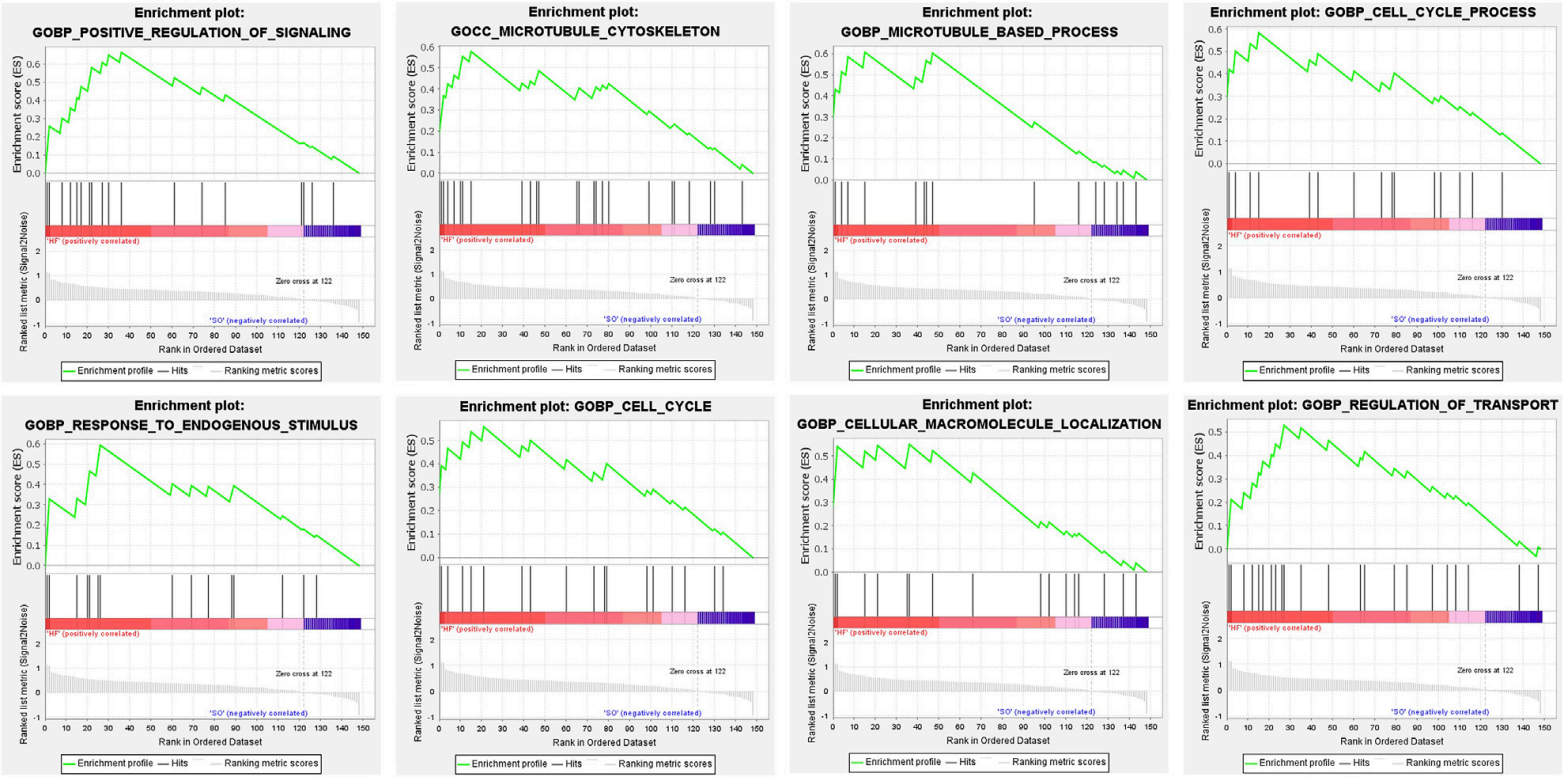
Gene set enrichment analysis. GSEA in HF group. NOM *p* < 0.05, FDR <25%.

To identify overall characteristics of post-infarction chronic heart failure, we performed gene set enrichment analysis through GSEA. Positive regulation of signaling, microtubule cytoskeleton, microtubule-based process, cell cycle process, response to endogenous stimulus, cell cycle, cellular macromolecule localization, and regulation of transport were significantly enriched in post-infarction chronic heart failure (Figure 3).

### Functional enrichment analysis of DPPs

**FIGURE 4:**
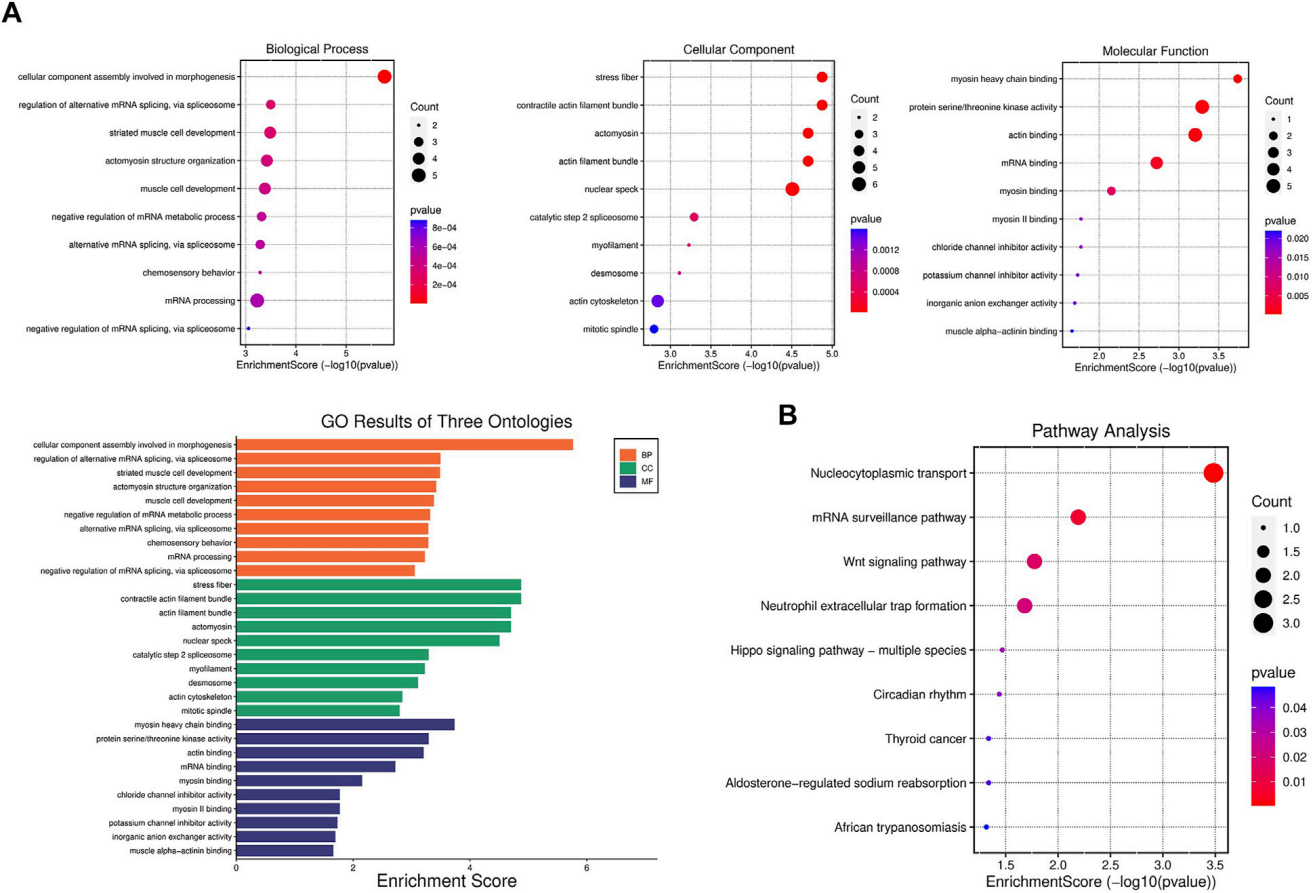
Gene set enrichment analysis of differentially expressed phosphorylated proteins **(A)** GO enrichment analysis of biological process, cellular component, and molecular function enriched by differentially expressed phosphorylated proteins (DPPs). **(B)** KEGG pathway enrichment analysis enriched by DPPs.

GO and KEGG pathways analysis were performed to explore the potential biological functions and pathways of DPPs in post-infarction chronic heart failure. The top 10 results of GO analysis and the top 9 results of KEGG pathways analysis were shown in the enrichment scatter plots. According to GO analysis, DPPs were significantly enriched in related processes including nuclear speck, and regulation of alternative mRNA splicing (Figure 4A). KEGG pathways analysis showed that DPPs were involved in nucleocytoplasmic transport, mRNA surveillance pathway, and Wnt signaling pathway (Figure 4B).

### Protein-protein interaction network construction and analysis

**FIGURE 5:**
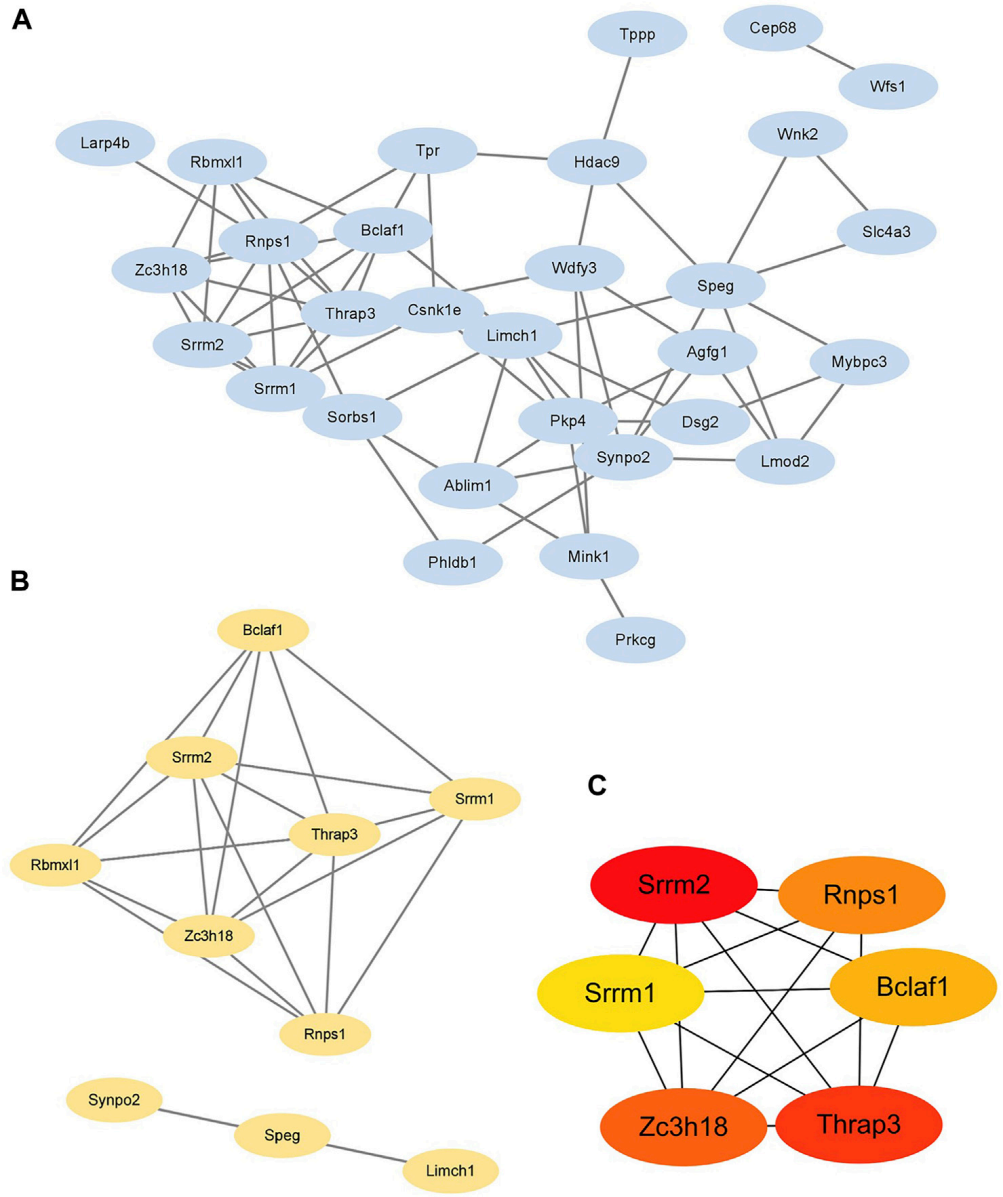
Identification of six hub differentially expressed phosphorylated proteins **(A)** The protein-protein interaction (PPI) network of 30 differentially expressed phosphorylated proteins (DPPs). **(B)** Two clusters of the PPI network. **(C)** Hub DPPs calculated based on the maximum neighborhood component (MNC), degree, and edge percolated component (EPC) algorithms.

The STRING database and Cytoscape 3.9.1 were applied to explore the interactions between DPPs. After removing the isolated DPPs, the PPI networks of DPPs were displayed including 30 nodes and 64 edges (Figure 5A). The PPI networks were divided into two clusters according to the MCODE algorithm scores (Figure 5B). The first cluster was composed of 7 DPPs (Bclaf1 Ser658, Srrm1 Ser634, Srrm2 Ser1181, Trap3 Ser243, Rbmxl1 Thr163, Zc3h18 Ser529, and Rnps1 Ser251). The second cluster was composed of 3 DPPs (Synpo2 Ser304, Speg Ser2448, and Limch1 Ser217). According to MNC, degree, and EPC algorithms, the top 6 DPPs were selected as the hub DPPs (Bclaf1 Ser658, Srrm1 Ser634, Srrm2 Ser1181, Trap3 Ser243, Zc3h18 Ser529, and Rnps1 Ser251; Figure 5C).

### Intersection of DPPs and thanatos apoptosis database

**FIGURE 6:**
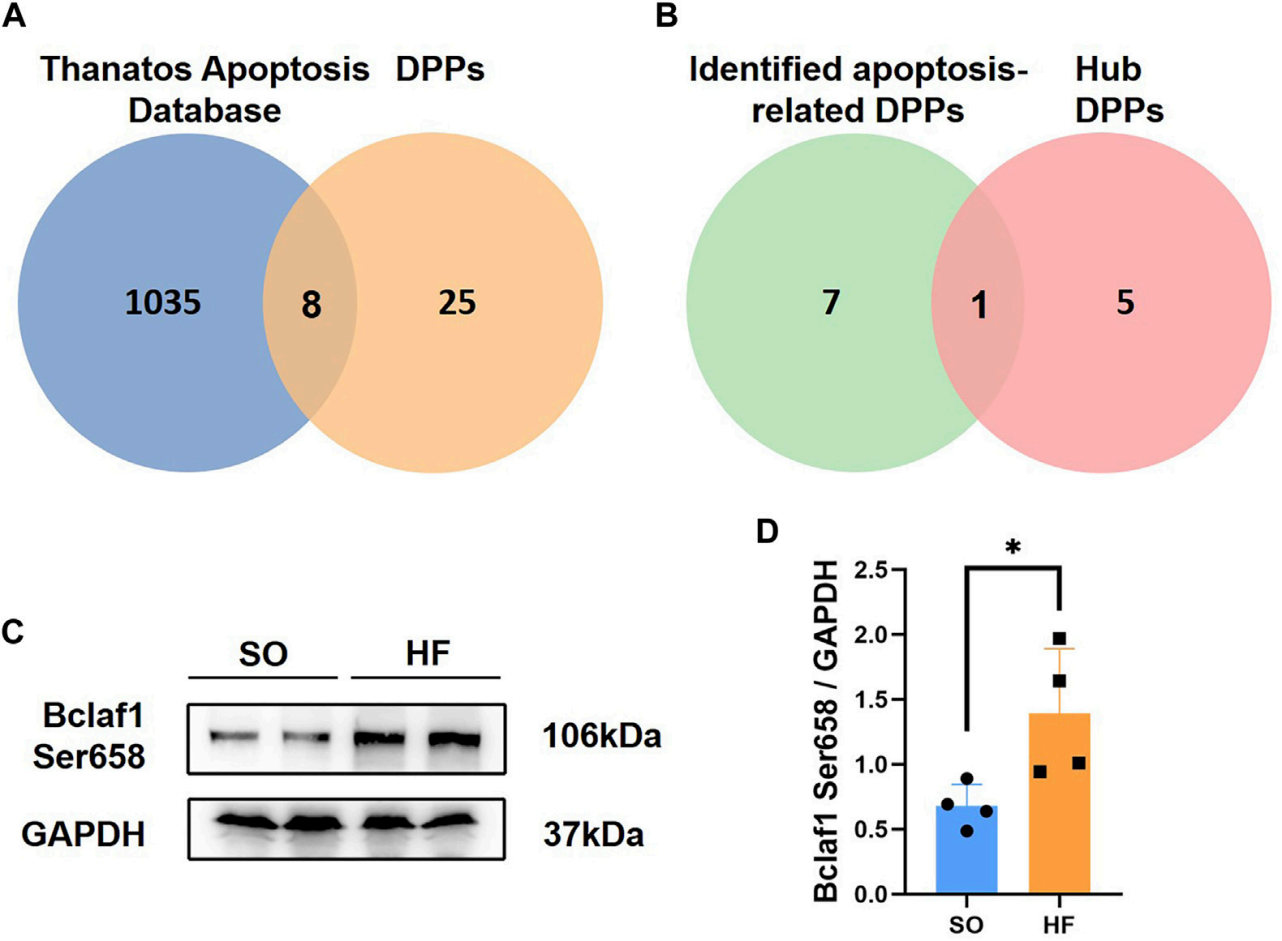
Intersection of differentially expressed phosphorylated proteins and Thanatos Apoptosis Database **(A)** Venn diagram of data in Thanatos Apoptosis Database and differentially expressed phosphorylated proteins (DPPs). **(B)** Venn diagram of identified apoptosis-related DPPs and hub DPPs. **(C,D)** Bclaf1 Ser658 expression in left ventricular from post-infarction chronic heart failure rats and sham operation (SO) rats.

Since cardiomyocyte apoptosis is critical for heart failure, the relationship between DPPs and cardiomyocyte apoptosis was analyzed. A total of 1,043 proteins associated with apoptosis were identified in Thanatos Apoptosis Database. As shown in Figure 6A. We obtained 8 proteins by taking the intersection of DPPs and Thanatos Apoptosis Database, including Bclaf1, Tpr, Wdfy3, Mybpc3, Csnk1e, Synpo2, Tppp, and Hdac9. After intersected with hub DPPs in PPI network, we found one hub DPP (Bclaf1 Ser658, Figure 6B).

### Left ventricular expression of Bclaf1 Ser658

To further validate our phosphoproteomic findings, we assessed protein level of Bclaf1 Ser658 with Western blotting from other post-infarction chronic heart failure/SO rats. Expression level of Bclaf1 Ser658 was increased post-infarction chronic heart failure rats (Figures 6C, D), which is consistent with the previous phosphoproteomic analysis.

### Predicted upstream kinases of DPPs in post-infarction chronic heart failure and validation

**FIGURE 7:**
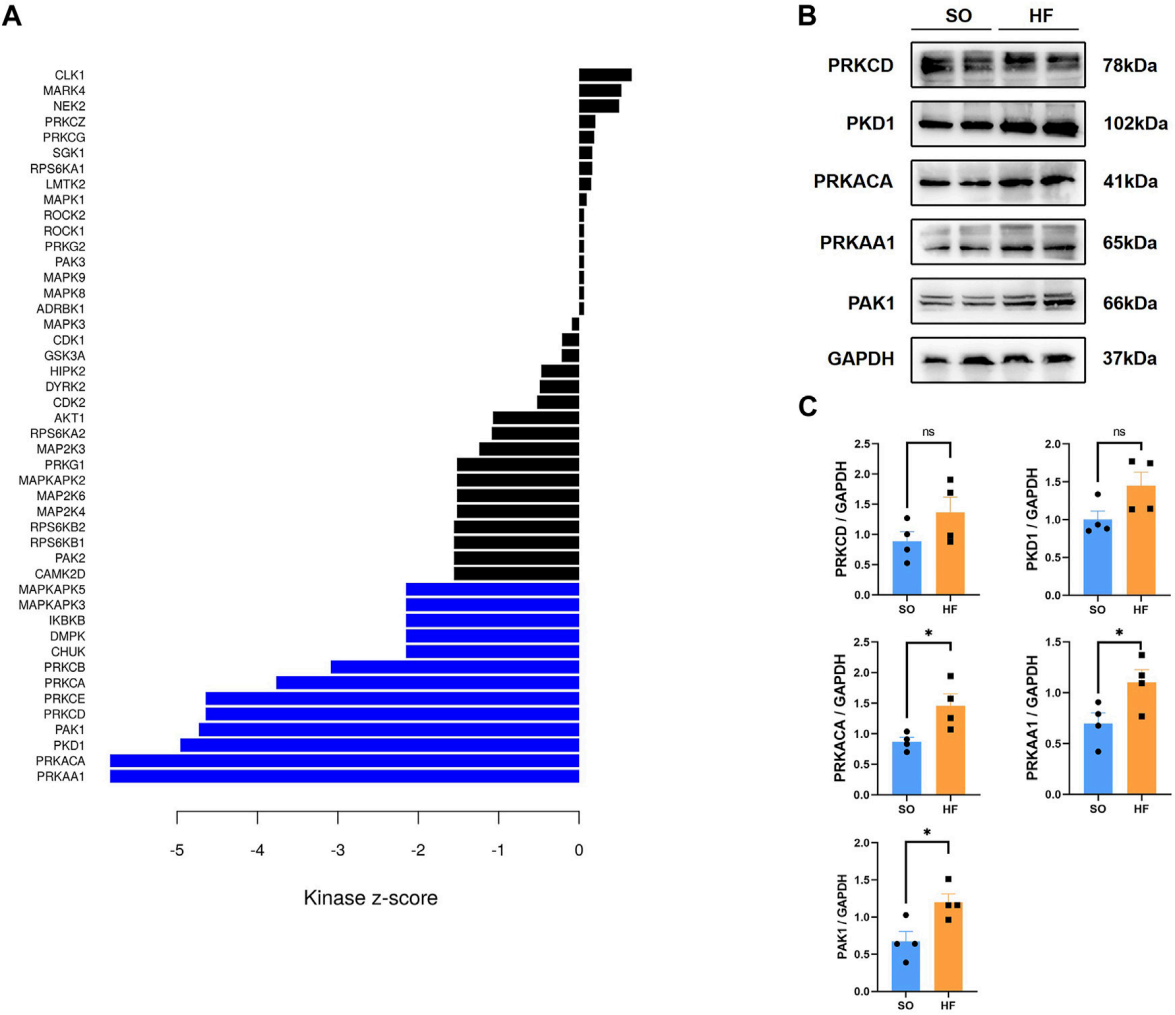
Kinome profiling from the phosphoproteomic data and validation **(A)** Bar plot of kinase-substrate enrichment analysis (KSEA) among up and downregulated phosphorylated proteins in post-infarction chronic heart failure rats and sham operation (SO) rats. Blue bars represent enrichment in the phosphor-sites that are upregulated in post-infarction chronic heart failure tissues relative to SO tissues. **(B,C)** Protein kinase AMP-activated A1(PRKAA1), protein kinase A catalytic α subunit (PRKACA), protein kinase D1 (PKD1), p21-activated kinase 1 (PAK1), and protein kinase C delta (PRKCD) expression in left ventricular from post-infarction chronic heart failure rats and SO rats.

Profiling of kinases can be useful to guide treatment of post-infarction chronic heart failure on the basis of phosphoproteomic data. Therefore, we performed kinome profiling based on KSEA app to predict active upstream kinases in post-infarction chronic heart failure. According to kinase-substrate relationships, we examined the phosphoproteomic data and performed phosphosite-based KSEA enrichment analysis. A total of 13 kinases were predicted to be significantly different in post-infarction chronic heart failure compared to SO (Figure 7A, Blue). The top 5 predict active upstream kinases were PRKAA1, PRKACA, PKD1, PAK1, and PRKCD. We then assessed protein level of the top 5 to predict active upstream kinases with Western blotting from other post-infarction chronic heart failure/SO rats. Expression levels of PRKAA1, PRKACA, and PAK1 were increased in post-infarction chronic heart failure rats (Figures 7B, C).

### Proteomic profiling of the LV in post-infarction chronic heart failure

**FIGURE 8:**
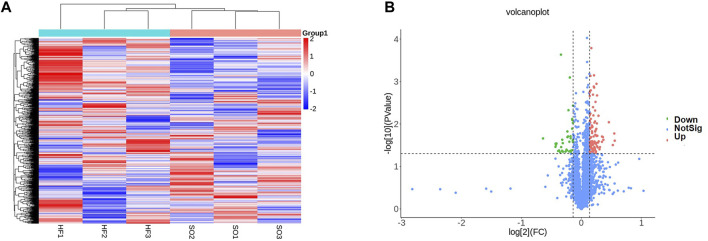
Proteomic profiling **(A)** Heat map comparison of differentially expressed proteins between the post-infarction chronic heart failure (HF) group and sham operation (SO) group. HF group compared to SO group: Red represents upregulated phosphorylated proteins and blue represents downregulated phosphorylated proteins. **(B)** Volcano plots displaying significantly differentially expressed proteins. Red dots represent the upregulated proteins and blue dot represents the downregulated proteins.

We then performed proteomic analysis of LV tissues. Among all 3,131 identified proteins, 129 proteins were differentially expressed between two groups (Figures 8A, B).

**FIGURE 9:**
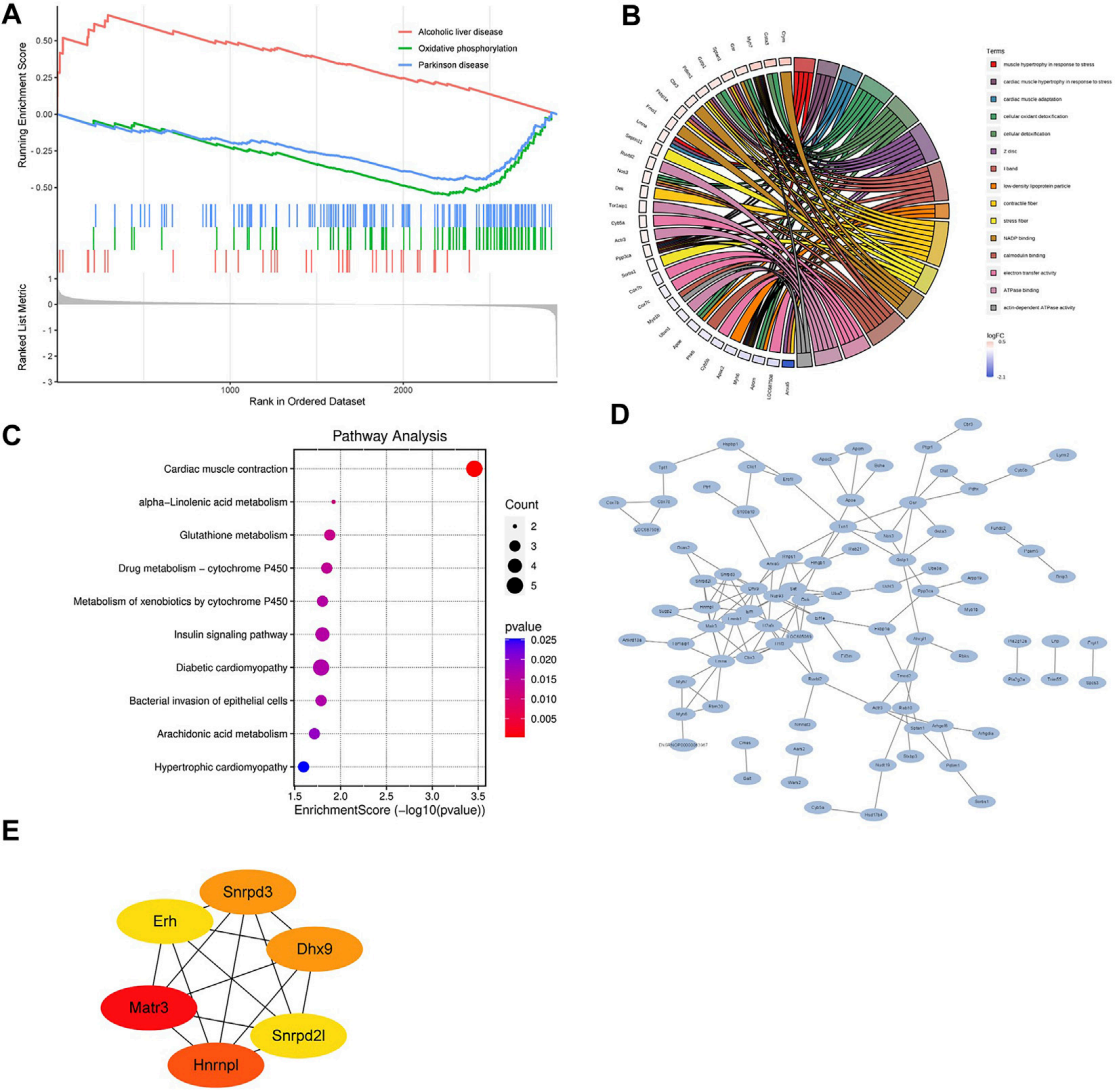
Bioinformatics analysis of differentially expressed proteins **(A)** Gene set enrichment analysis. NOM *p* < 0.05, FDR <25%. **(B)** Relationship between the top 15 significant GO terms and enriched proteins. **(C)** KEGG pathway enrichment analysis enriched by differentially expressed proteins. **(D)** The protein-protein interaction (PPI) network of differentially expressed proteins. **(E)** Hub DPPs differentially expressed proteins based on the maximum neighborhood component (MNC), degree, and edge percolated component (EPC) algorithms.

Next, we performed gene set enrichment analysis through GSEA to identify overall characteristics of post-infarction chronic heart failure (Figure 9A). GSEA showed several significant enriched gene sets involved in cardiac metabolism in the LV, including oxidative phosphorylation, metabolism of xenobiotics by cytochrome P450, and hlycerolipid metabolism (Supplementary Table S2).

GO and KEGG pathways analysis were performed subsequently to explore the potential biological functions and pathways of differentially expressed proteins in post-infarction chronic heart failure. Figure 9B showed the relationship between the top 15 significant GO terms and enriched proteins. Z disc, I band, contractile fiber, calmodulin binding, and electron transfer activity were the top 5 significant terms, which were correlated with depressed cardiac contractility. KEGG pathways analysis showed that differentially expressed proteins were involved in cardiac muscle contraction, alpha-Linolenic acid metabolism, glutathione metabolism, drug metabolism-cytochrome P450, metabolism of xenobiotics by cytochrome P450, insulin signaling pathway, diabetic cardiomyopathy, bacterial invasion of epithelial cells, arachidonic acid metabolism, hypertrophic cardiomyopathy (Figure 9C).

Finally, we built the PPI networks of differentially expressed proteins (Figure 9D). According to MNC, degree, and EPC algorithms, the top 6 differentially expressed proteins were selected as the hub differentially expressed proteins (Matr3, Hnrnpl, Snrpd3, Dhx9, Erh, and Snrpd2l; Figure 9E).

## Discussion

Investigation and analysis of PTMs are essential to understand the function of many proteins in pathophysiological processes. Protein phosphorylation serves as a critical regulatory mechanism for distribution, trafficking, and function of modified proteins (Wang et al., 2014). It is also noteworthy that the predominant PTM in heart is phosphorylation (Van Eyk, 2011; Smith and White, 2014). In myocardial ischemia/reperfusion (I/R) injury process, hyperphosphorylated adenosine monophosphate-activated protein kinase resulted (MAPK) in decreased pro-inflammatory cytokines and inhibited cellular apoptosis and inflammation (Zhang et al., 2020). In hypertensive heart disease, factor-alpha-related protein 9 (CTRP9) decreased phosphorylation levels of phosphatidylinositol 3-kinase (PI3K), protein kinase B (Akt), and mammalian target of rapamycin (mTOR) proteins, which led to reduced vascular endothelial cell injury (Pan et al., 2022). However, potential phosphorylation-related regulatory mechanism underlying post-infarction chronic heart failure remains unclear.

In this study, we examined the global quantitative phosphoproteomics and proteomics of LV tissues from post-infarction chronic heart failure rats and SO. Based on phosphoproteomic, proteomics data, and external databases, we investigated and revealed phosphorylation characteristics, pathway alterations, and potential therapeutic targets in post-infarction chronic heart failure.

The functional enrichment analysis supports the existence of an hyperactivation of nucleocytoplasmic transport process in post-infarction chronic heart failure. Nucleocytoplasmic transport process includes bidirectional transport between the nucleus and the cytoplasm, which is critical for the survival of eucaryotic cells (Beck and Hurt, 2017). Previous studies showed expressions of nucleocytoplasmic transport-related genes were altered in heart failure (Molina-Navarro et al., 2013). For example, mRNA level of XPO1, an important exportin of nuclear (Hutten and Kehlenbach, 2007), is closely related with robust left ventricular function parameters in patients with ischemic heart failure. In this study, we detected several proteins related to nucleocytoplasmic transport process that were hyperphosphorylated in post-infarction chronic heart failure. These included Srrm2 and Rnps1. Tanaka et al. reported that phosphorylation of Srrm2 increased the translocation of Srrm2 to the cytoplasm and affected the RNA splicing of synapse genes, which led to cognitive impairment of Alzheimer’s disease (Tanaka et al., 2018). Rnps1, one of the essential components of exon junction complex, was reported to have an ATP-regulated mechanism (Wagner et al., 2004). However, there is no single study on functions and mechanisms of phosphorylation of Srrm2/Rnps1 in heart failure. Since protein phosphorylation can alter the structure and activity of protein, we speculated that hyperphosphorylated Srrm2/Rnps1 might exacerbate post-infarction chronic heart failure through facilitating cargo export from nuclear pore. These 2 hyperphosphorylated proteins are also integral to the mRNA surveillance pathway, pointing towards a possible alteration in this pathway. Gene set enrichment and GO analysis also showed significantly enriched terms including cell cycle process, regulation of transport, nuclear speck, and regulation of alternative mRNA splicing. Results of Gene set enrichment and GO analysis correlated well with each other. These findings provide novel altered pathways and potential therapeutic targets in post-infarction chronic heart failure treatment.

Apoptosis is one of the typical characteristics of cardiac remodeling following MI (Sun et al., 2000). In this study, we intersected DPPs with Thanatos Apoptosis Database and identified Bclaf1 Ser658 as a potential critical phosphorylated protein of apoptosis in post-infarction chronic heart failure. Interestingly, the function of Bclaf1 has been enigmatic. Bclaf1 was initially considered to be a protein partner of adenovirus bcl-2 homologue E1B19K (Zhou et al., 2014). Previous work has revealed that Bclaf1 served as an inducer of apoptosis and transcription repression factors (McPherson et al., 2009). However, some recent research by Zhang et al. reported that Bclaf1 has been associated with the anti-apoptotic program of TNF and absence of Bclaf1 would lead to severe apoptosis and pyroptosis in TNF-induced small intestine injury (Zhang et al., 2022). In this study, Bclaf1 was phosphorylated at Ser658 in post-infarction chronic heart failure. Enrichment analysis coupled with PPI network analysis showed a tight association between Bclaf1 Ser658 and nucleocytoplasmic transport process. Therefore, we speculate that the phosphorylation of Bclaf1 at Ser658 would promote cardiomyocyte apoptosis after MI through nucleocytoplasmic transport process. Recently, Zhang et al. reported that the long noncoding RNA Cardiac Injury-Related Bclaf1-Inhibiting LncRNA (lncCIRBIL) could directly bind to Bclaf1 and inhibit its nuclear translocation, which led to a protective effect in cardiac I/R injury (Zhang et al., 2021b). This is consistent with our analysis above. However, the underlying mechanisms still needs to be further explored and validated.

Upstream kinases of DPPs were predicted based on KSEA app. The activities of PRKAA1, PRKACA, and PAK1 were significantly enhanced in post-infarction chronic heart failure. Researchers can investigate the effects of targeted therapies based on inhibitors of these predicted kinases. However, the underlying mechanism still need to be further explored and validated.

Furthermore, proteomic analysis of LV tissues showed marked changes in protein expression related to cardiac contractility and metabolism. Proteins related to glutathione metabolism significantly decreased, which suggested that one of the major endogenous factors of the antioxidant defence for the inactivation of reactive oxygen species was impaired. Based on PPI network construction and modules analysis, we finally found 6 hub differentially expressed proteins, including Matr3, Hnrnpl, Snrpd3, Dhx9, Erh, and Snrpd2l. The expression of Matr3 is related to cardiac muscle diseases (Savarese et al., 2020). Hnrnpl might serve as a potential therapeutic target for WW domain-containing E3 ubiquitin ligase (WWP2) -mediated anoxia/reoxygenation-induced cardiomyocyte dysfunction (Song et al., 2022). Dhx9, Snrpd3, Erh, and Snrpd2l might be novel potential therapeutic targets for heart failure.

To apply the results of the present study in clinical practice of prevention and treatment of post-infarction chronic heart failure, further studies need to overcome several drawbacks of the present study. Firstly, samples for phosphoproteomic and proteomic analysis were taken from animal models and sample size was too small to detect small differences between groups. Secondly, we only performed the validation of one potential critical phosphorylated protein of apoptosis and top 5 predicted upstream kinases of DPPs. Further studies based on our research need to validate more DPPs, upstream kinases, and differentially expressed proteins.

## Conclusion

In conclusion, the present study marked phosphoproteomics and proteomics changes in post-infarction chronic heart failure. Bclaf1 Ser658 might play a critical role in apoptosis in heart failure. PRKAA1, PRKACA, and PAK1 might serve as potential therapeutic targets for post-infarction chronic heart failure. Further studies are needed to validate our results and explore potential mechanisms.

## Data Availability

The data presented in the study are deposited in the ProteomeXchange Consortium (http://proteomecentral.proteomexchange.org) via the iProX partner repository, accession number PXD042843.
