# Supplementary figures and images for: Phosphoproteomic and proteomic profiling in post-infarction chronic heart failure
Source: Front Pharmacol. 2023 Jun 19;14:1181622. doi: 10.3389/fphar.2023.1181622 (PMC10315476; doi:10.3389/fphar.2023.1181622)

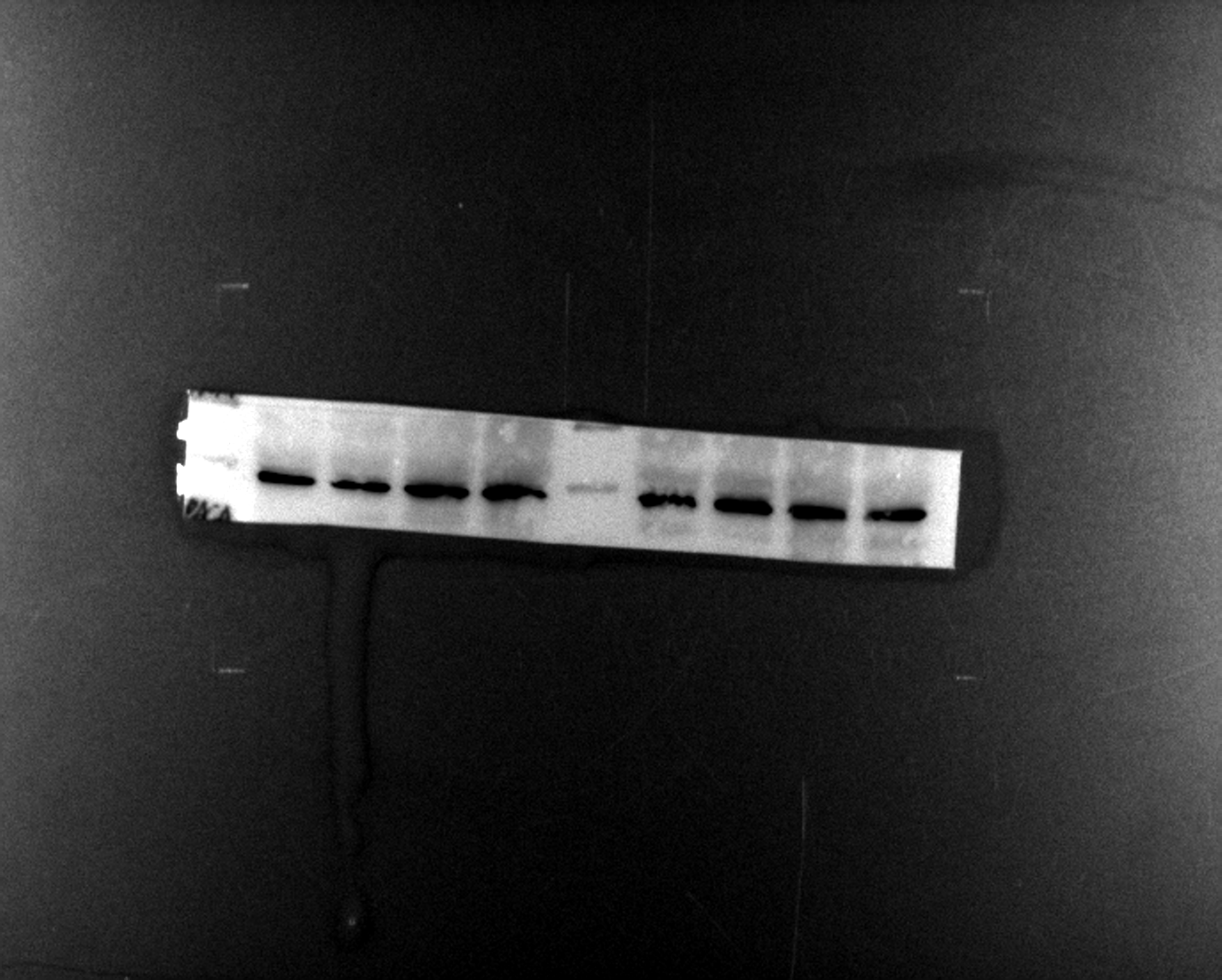

Supplement: Supplementary file 2 [file Image6.TIF]

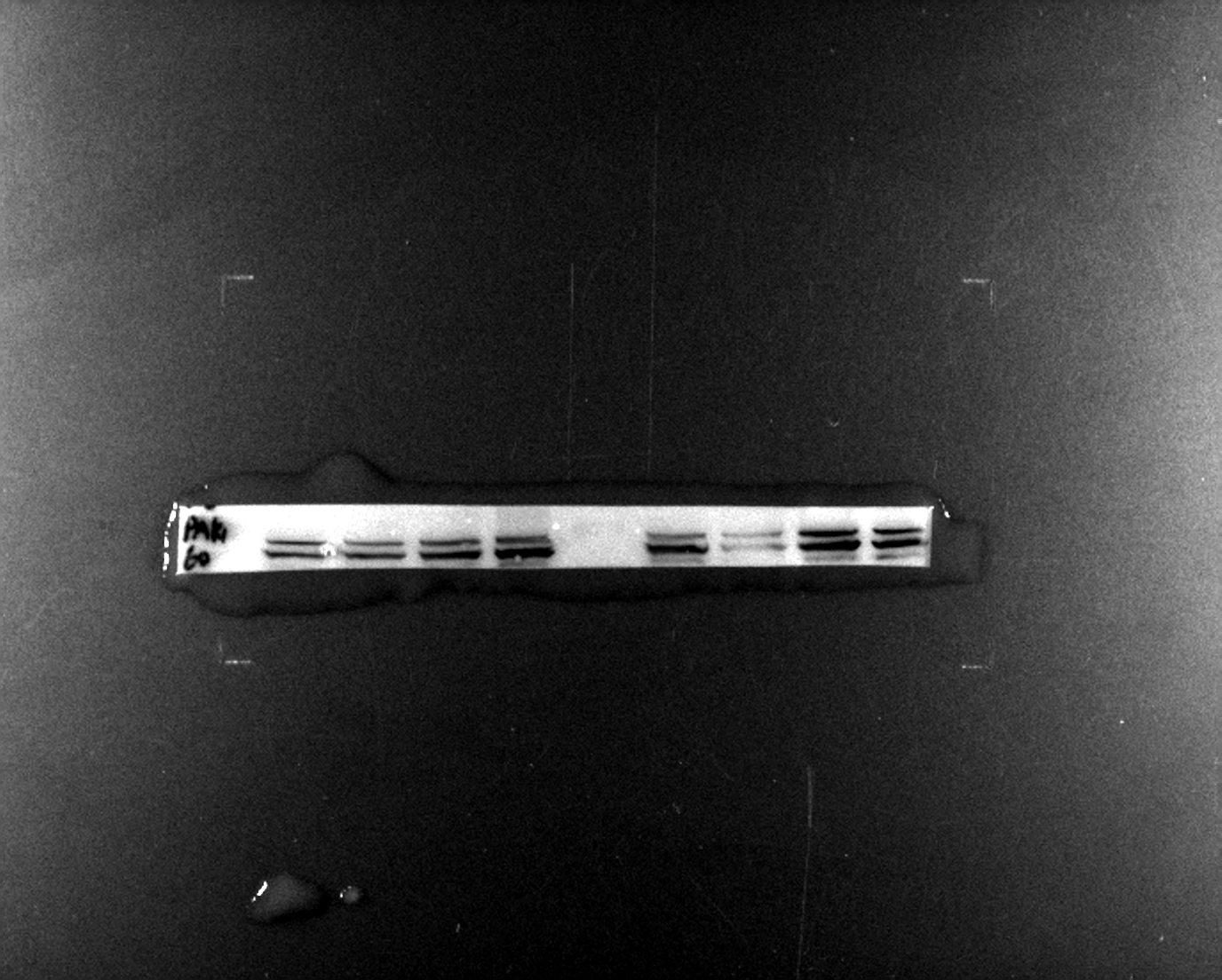

Supplement: Supplementary file 3 [file Image3.TIF]

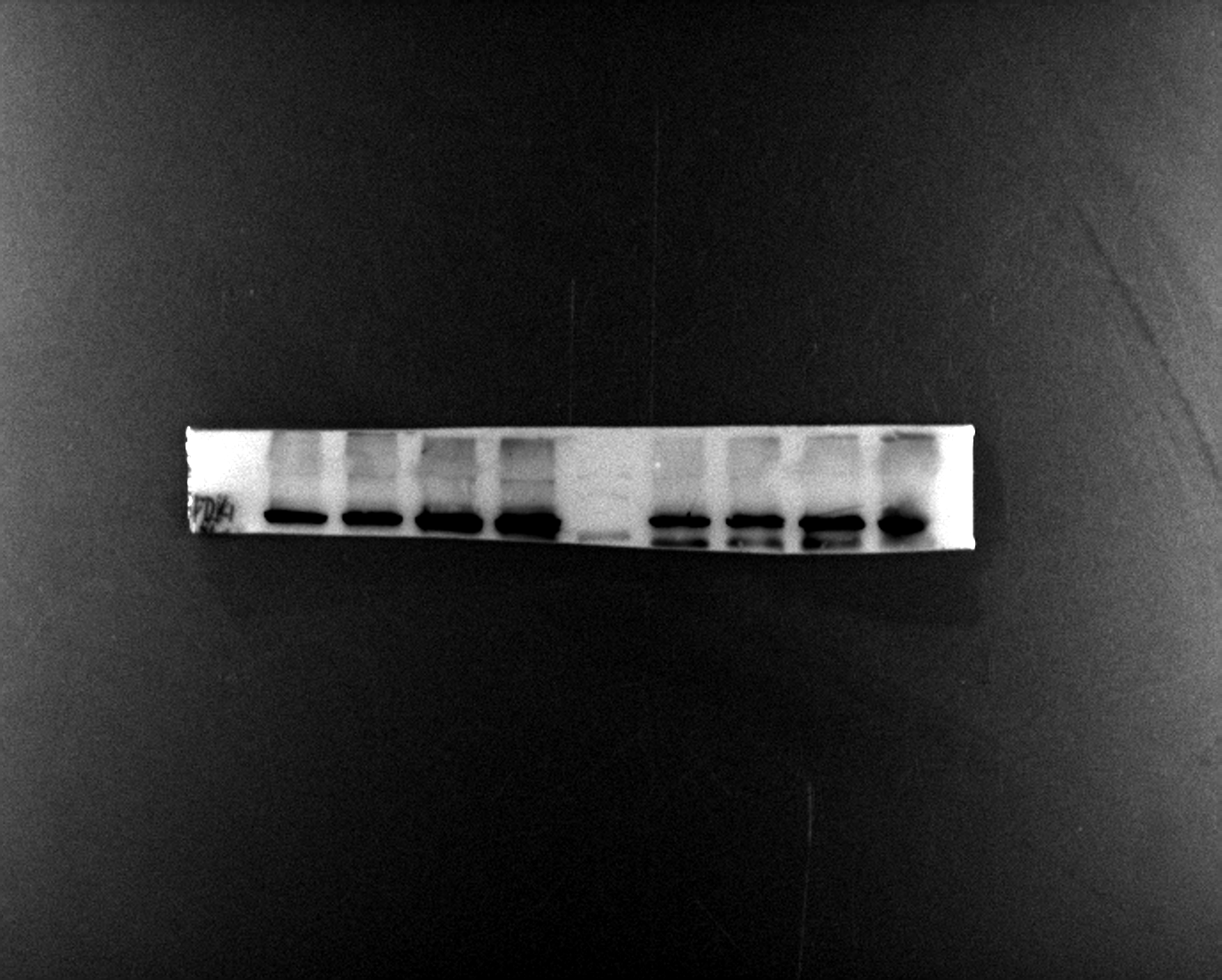

Supplement: Supplementary file 4 [file Image4.TIF]

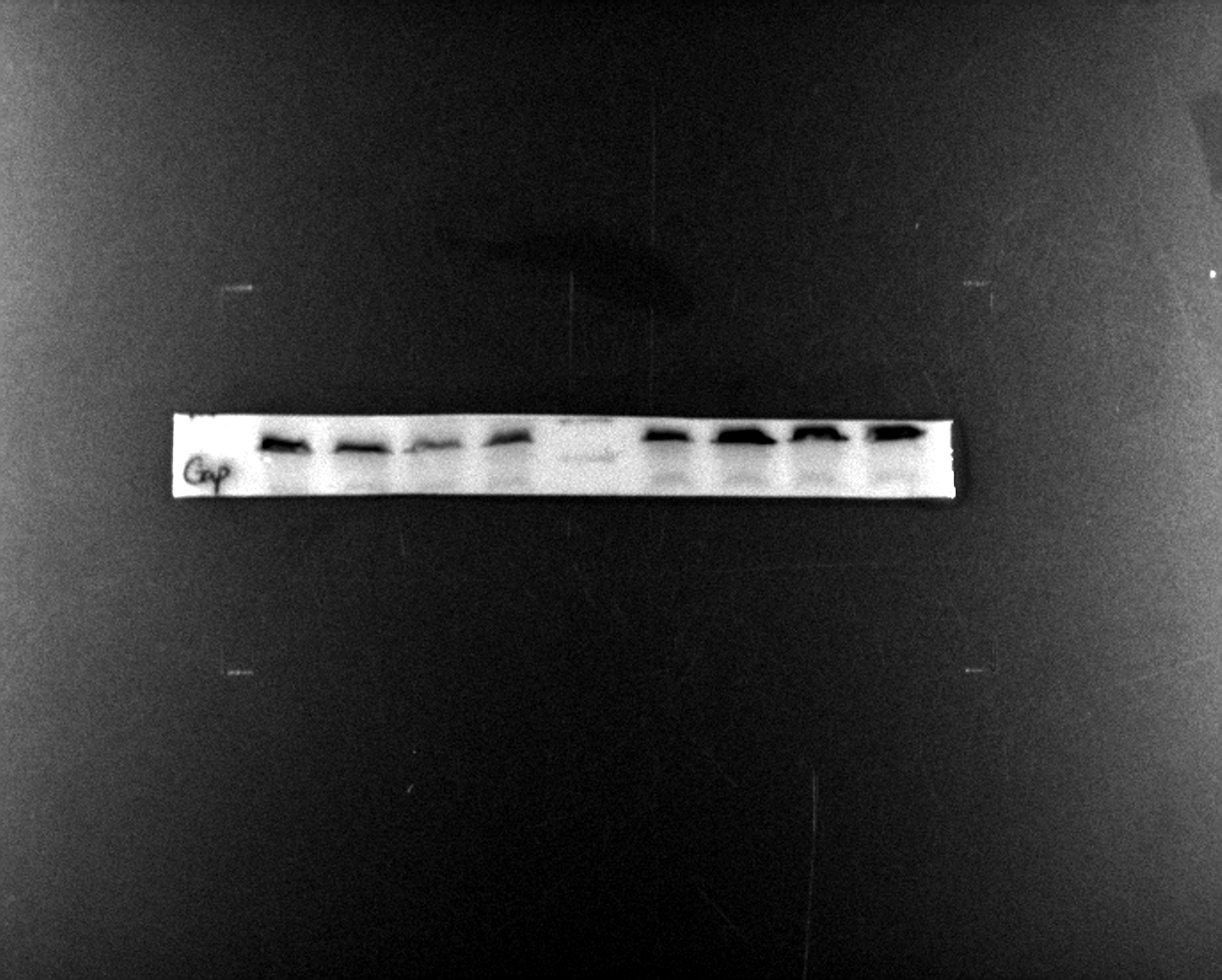

Supplement: Supplementary file 5 [file Image2.TIF]

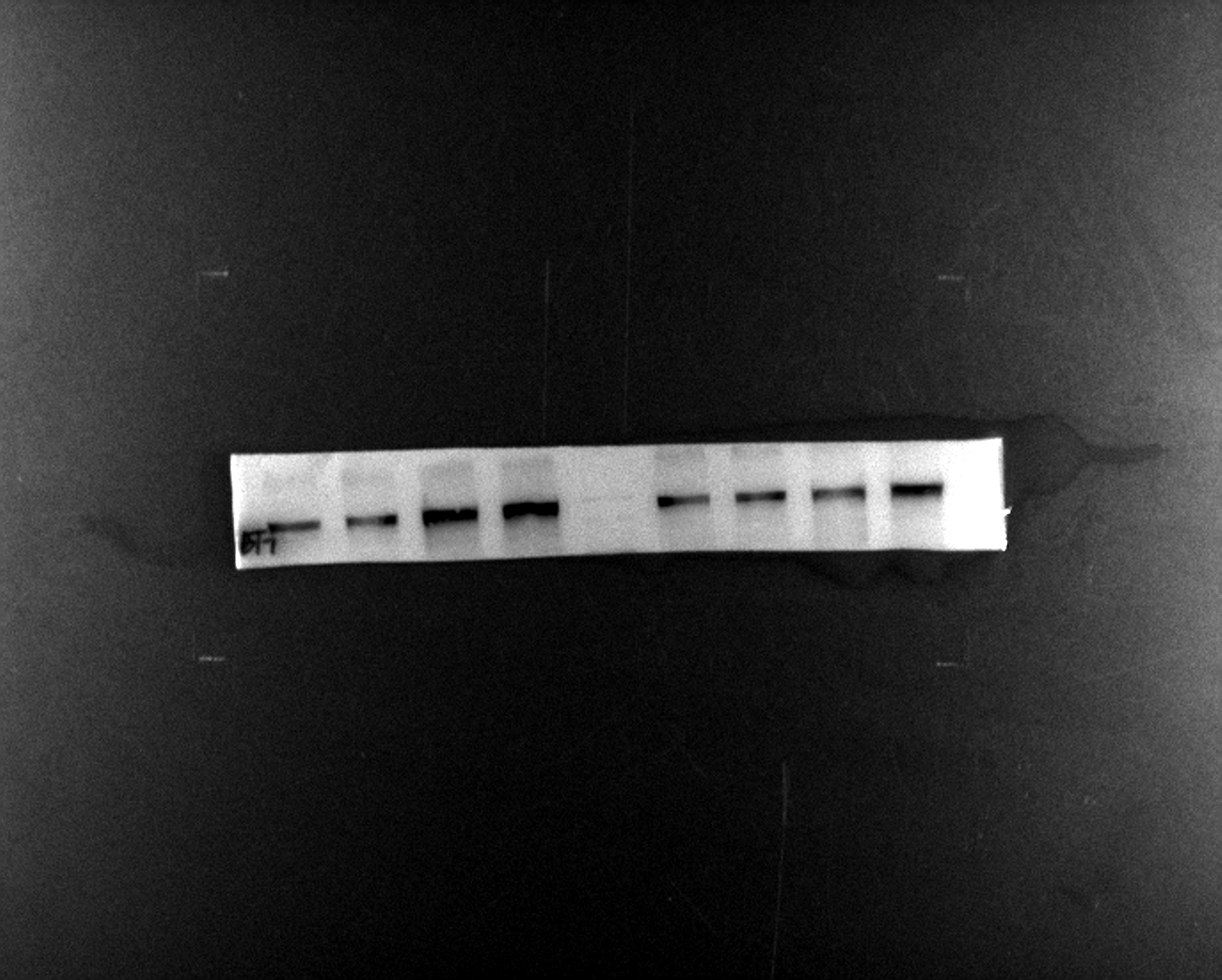

Supplement: Supplementary file 6 [file Image1.TIF]

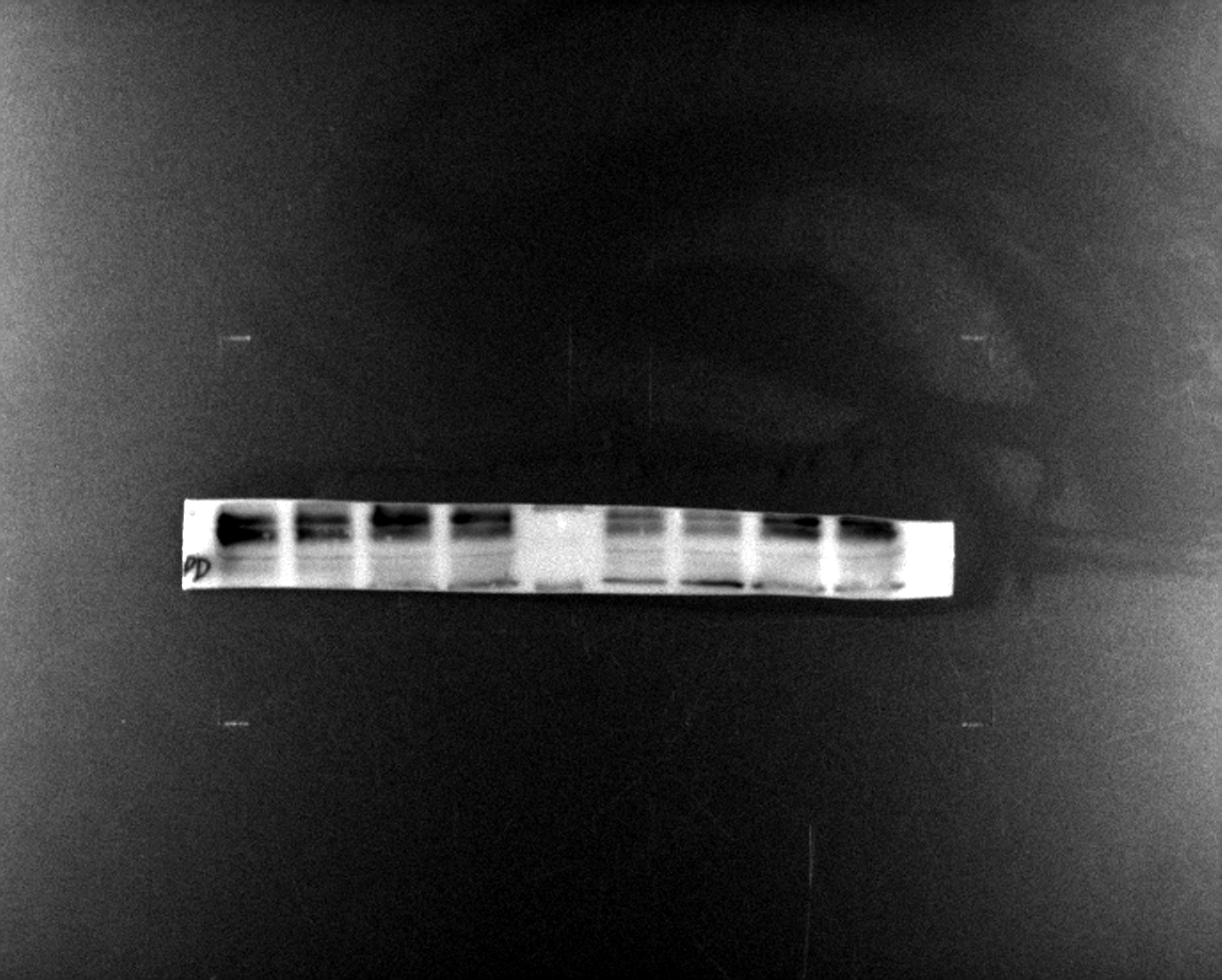

Supplement: Supplementary file 7 [file Image7.TIF]

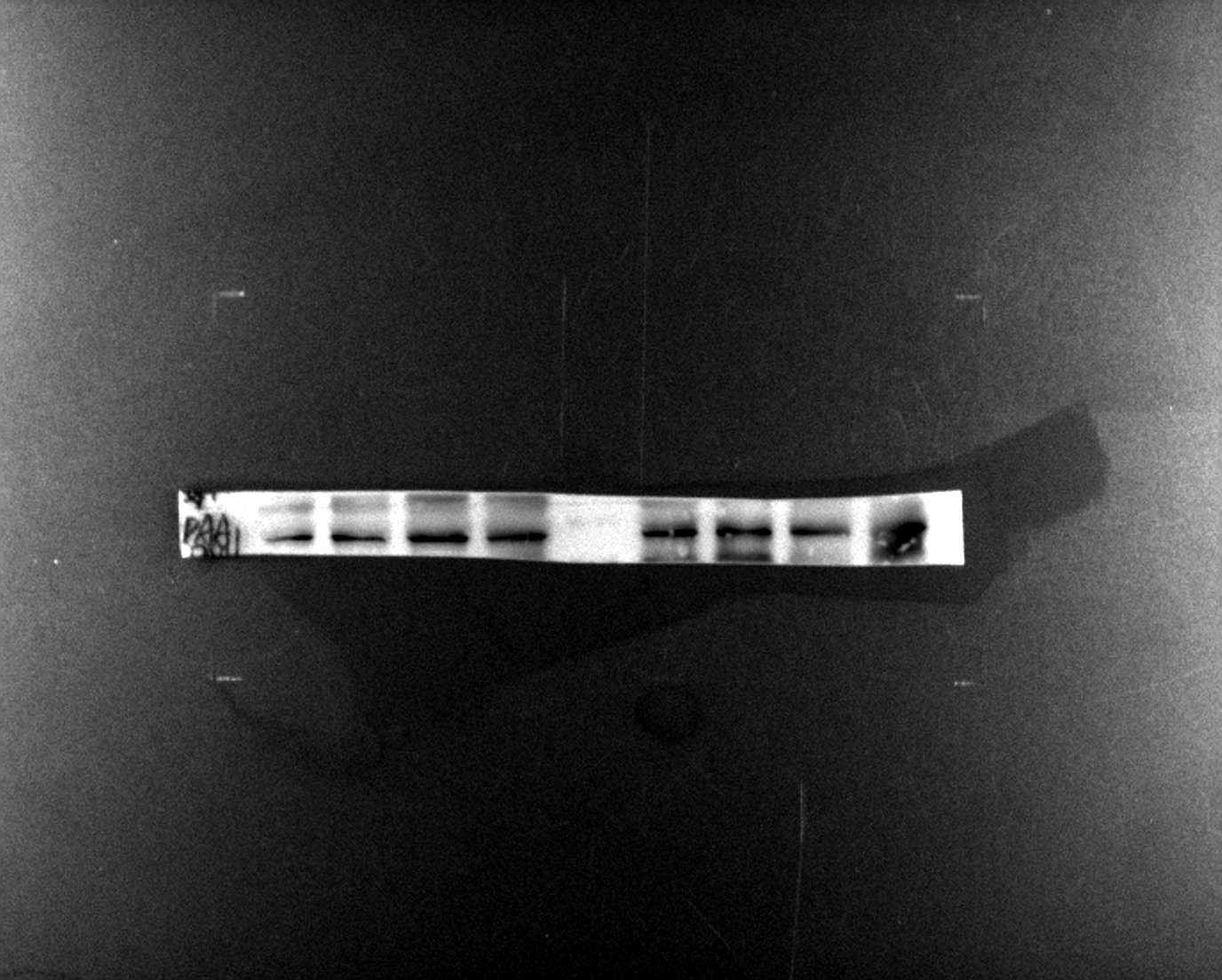

Supplement: Supplementary file 9 [file Image5.TIF]
